# High-Stable Electric Field Integrated Optical Sensor Based on Reduced Lithium Niobate

**DOI:** 10.3390/s26051619

**Published:** 2026-03-04

**Authors:** Aleksei Sosunov, Artem Shipitsin, Mikhail Zhitkov, Anton Kuznetsov, Andrey Kosberg, Anton Zhuravlev, Andrey Lutsenko, Victor Krishtop, Anatoliy Mololkin

**Affiliations:** 1 Perm State University, 614990 Perm, Russia; 2 Perm Scientific Industrial Instrument-Making Company, 614990 Perm, Russia; 3 Perm National Research Polytechnic University, 614990 Perm, Russia; 4 National University of Science and Technology “MISIS”, 119049 Moscow, Russia

**Keywords:** reduced lithium niobate, integrated optics, electric field sensors, Michelson interferometer, pyroelectric effect, optical instability, device calibration

## Abstract

Integrated optical devices based on lithium niobate (LN) are pivotal in modern navigation systems, telecommunications, and sensing technologies. However, their practical implementation is critically limited by temperature-dependent and long-term operational instability, primarily attributed to the pyroelectric effect inherent in LN. This study addresses this challenge by investigating thermally reduced lithium niobate as a material platform to enhance the stability of integrated optical circuits, with a focus on integrated optical electric field sensors (IOES). We present the fabrication and comprehensive characterization of an IOES based on a Michelson interferometer design. Key performance metrics including optical loss, free spectral range, electro-optical sensitivity, and optical path difference were systematically evaluated. Notably, under normal climatic conditions, the optical path difference of the IOES demonstrated exceptional stability when subjected to an applied voltage ranging from 0 to 5 V, with no observable drift over time. Calibration of the IOES revealed a predominantly linear response, although a third-degree polynomial model provided a more precise fit to the experimental data. The minimum relative error achieved during calibration was 0.47%, underscoring the high accuracy of the device. Our results establish thermally reduced LN as a promising material platform for next-generation integrated optical devices. By mitigating the pyroelectric effect, this approach enables significant improvements in the long-term stability of IOES and other LN-based photonic components. These findings open avenues for the reliable deployment of integrated optical systems in demanding applications where environmental stability is paramount.

## 1. Introduction

Lithium niobate (LN) is a cornerstone material in integrated photonics [1], owing to its exceptional combination of properties: a broad transparency window (0.3–5 μm), a high Curie temperature (1140 °C), excellent optical homogeneity (refractive index variation ∆*n* ≈ 10^−5^ over a 10 mm scale at 1550 nm), a strong electro-optic coefficient (*r*_33_ = 32 pm·V^−1^), and a significant nonlinear optical coefficient (*d*_33_ = −33 pm·V^−1^). These attributes enable the fabrication of both low-contrast [2] and high-contrast [3] waveguides with ultra-low propagation losses (<0.2 dB/cm) in the C-band (λ = 1550 nm).

The change in the refractive index of LN under an applied electric field (E-field) is generally described by a third-rank tensor [4]:

(1) $${\left(\frac{1}{∆n}\right)}_{ij}=\sum_{k}r_{ijk}E_{k},$$

where *E_k_* are the components of the applied E-field and *r_ijk_* are the third-rank electro-optic tensor (for LN, the nonzero elements correspond to its 3 m crystal symmetry). To utilize the maximum electro-optic coefficient *r*_33_ of LN, the applied E-field must be aligned with the polar Z-axis. The resulting change in the extraordinary refractive index, ∆*n_e_*, can then be described as:

(2) $$∆n_{e}=-\frac{1}{2}n_{e}^{3}r_{33}E_{z}.$$

In 1980, Bulmer et al. first demonstrated the feasibility of electric field detection using an integrated optical E-field sensor (IOES) with asymmetric arms based on LN [5]. Scientific and industrial interest in this area remains high. Numerous approaches have since been developed to fabricate IOES for detecting high (~kV/m) and low (~μV/m) electric fields [6,7,8,9], as well as DC and AC voltages [10]. Other developed configurations include sensors based on Bragg gratings in waveguides [11], LN-based IOES with tapered antenna arrays [12,13], Mach–Zehnder interferometers with optical delay-modulation [14] or dipole patch antennas [15], Mach–Zehnder interferometers with asymmetric straight waveguides [16], domain-inverted LN waveguides [17], and 2D and 3D electric field sensors [18,19]. Each approach offers distinct advantages and potential for practical application.

However, a major challenge for IOES remains their long-term and temperature-dependent instability [20]. This instability is evident across all LN-based integrated optical devices, including IOES [21,22], multifunctional integrated optical circuits [23,24], and Mach–Zehnder modulators [25,26,27]. The primary contributing factor is the pyroelectric effect in LN, which leads to the formation of electrical inhomogeneities on the LN surfaces and a depolarizing electric field [28]. Consequently, ensuring the stability of integrated optical devices is a critical challenge for their reliable practical application and long-term operation.

In this work, we present, to the best of our knowledge, the first study of an IOES based on thermally reduced lithium niobate, a material characterized by a significantly suppressed pyroelectric effect due to its enhanced electrical conductivity [29].

The aim of this work is to reduce the instability of an IOES by eliminating the pyroelectric effect in the LN substrate. The paper is structured as follows: (1) fabrication and characterization of the IOES based on reduced LN; (2) measurement of the optical stability of the IOES under normal ambient conditions; (3) calibration of the IOES at three different temperatures and evaluation of the associated error; and (4) a summary and conclusion regarding the prospects and future challenges of this technology.

## 2. Materials and Methods

### 2.1. Reduced Lithium Niobate

**Figure 1:**
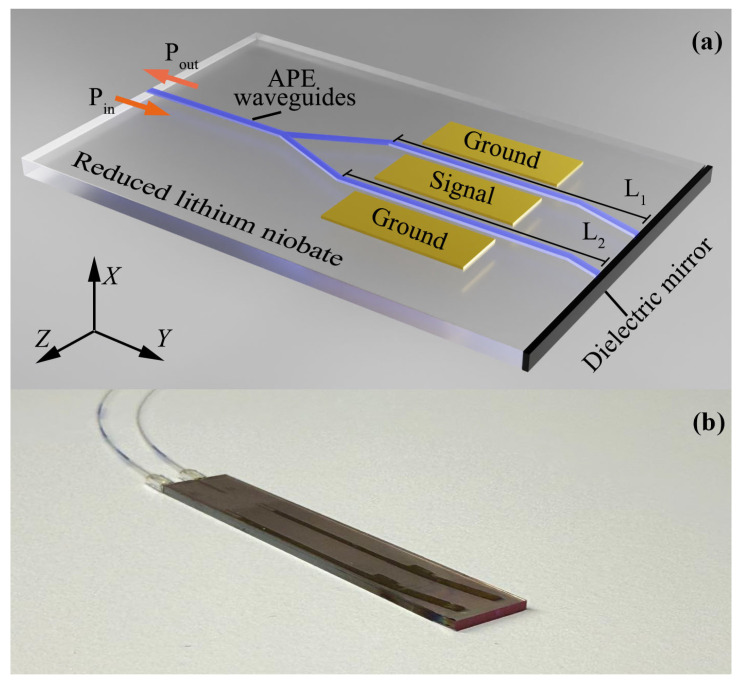
IOES based on reduced LN: scheme of IOES (**a**) and physical image of two IOES (**b**).

We used a commercially available, double-side polished, 1-mm-thick, *X*-cut LN wafer (4-inch diameter, Fomos-Materials, Russia) as the substrate (Figure 1b). The reduced LN wafer was obtained via thermal annealing at 700 °C for 2 h under a vacuum of 10^−1^ Pa. The resulting material exhibits a specific electrical resistance of 10^11^ Ω·cm and a transmittance of at least 75% in the 1500–1600 nm range.

### 2.2. Integrated Optical E-Sensor

- Theory

The IOES was implemented on the reduced LN substrate using a Michelson interferometer configuration [30]. The optical signal is split into a 1:1 ratio by a Y-splitter, directing it into two asymmetric arms with a length difference of 200 μm. The signal is then reflected from a highly efficient dielectric mirror and propagates back through the waveguides in the opposite direction. This design doubles the effective electro-optical interaction length while maintaining a compact chip footprint. A key feature of our IOES is the use of a highly reflective TiO_2_ dielectric mirror deposited onto the end-face of the LN substrate (Figure 1).

The operating principle of the IOES is based on the dependence of the output optical power *P_out_* on the phase difference Δ*φ* between the interferometer arms, which can be described by the following general equation:

(3) PoutPin=Re−2α(L1+L2)(1 + cos(∆φ)),

where *P_in_* is the input optical power, *R* is the dielectric mirror reflectivity, *α* is the optical insertion loss coefficient of the waveguides, and *L*_1_ and *L*_2_ are the lengths of the first and second waveguide, respectively.

The total phase difference Δ*φ* in the Michelson interferometer comprises two components: the intrinsic optical path difference between the arms Δ*φ*′ and the phase shift induced by the applied electric field E via the linear electro-optic (Pockels) effect Δ*φ*″:

(4) $$∆\varphi =\;∆\varphi^{\prime }\left(∆L\right)+\;∆\varphi^{\prime \prime }\left(E\right),$$

(5) $$∆\varphi^{\prime }(∆L)=\frac{4\pi n_{e}\left(L_{2}-L_{1}\right)}{\lambda },$$

(6) $$∆\varphi^{\prime \prime }\left(E\right)=\frac{4\pi \Gamma n_{e}^{3}r_{33}E_{z}}{\lambda },$$

where *λ* is the optical wavelength, Δ*L* = *L*_2_ − *L*_1_ is the optical path difference, and Γ is the overlap integral between the optical mode and the applied electric field.

Finally, accounting for the double pass of light, the total phase difference of the interferometer is given by:

(7) $$∆\varphi =\frac{4\pi }{\lambda }\left(n_{e}∆L+\Gamma n_{e}^{3}r_{33}E_{z}\right).$$

B. Manufacturing

Single-mode optical waveguides for 1550 nm operation were fabricated using a standard lift-off process with positive photoresist. A 600-nm-thick SiO_2_ layer was subsequently deposited via magnetron sputtering Caroline D12A1 (Russia) to serve as a protective hard mask.

The waveguides were then formed using the annealed proton-exchange (APE) technique [2]. This method is advantageous as it allows for low-temperature processing (~400 °C) and preserves the polarization state of the guided light (polarization-maintaining, PM).

The end-faces of the LN substrate were polished to optical quality using an OptiPrep™ polishing system. A highly reflective dielectric mirror, consisting of a 1.0 μm-thick TiO_2_ layer with a reflectivity of 99%, was deposited on one polished end-face via thermal evaporation Satisloh MC380H (USA).

Finally, metal electrodes (Au, 500 nm thick, 25 mm long) were patterned and deposited using the same magnetron sputtering system (Caroline D12A1, Russia).

For optical characterization, the APE waveguide was aligned to a PM fiber using micro-translators. A permanent splice was achieved by securing the fiber in a U-groove module and applying a UV-curable adhesive for fixation.

### 2.3. E-Sensor Characterization

The electro-optic sensitivity of the IOES based on reduced LN was characterized by measuring the wavelength shift ∆*λ* of an interference minimum in response to an applied external electric field. The sensitivity *S* was calculated using the relation:

(8) $$S=\frac{∆\lambda }{∆V},$$

where Δ*V* is the applied voltage. The measurement was performed using an optical spectrum analyzer Yokogawa AQ6370D (Japan). The shift in the interferometer’s spectrum was recorded across the 1500–1600 nm wavelength range while the applied voltage was swept from 0 to 25 V in increments of 0.5 V. The input (*P_in_*) and output (*P_out_*) optical signals were separated using a 1550 nm, 3-port, polarization-insensitive optical circulator.

The spectral characteristics of the IOES—specifically its reflection spectrum and free spectral range (FSR)—were measured using an Astro A313 (Russia) optical interrogator over the same 1500–1600 nm wavelength range.

The long-term stability of the IOES was investigated by monitoring the temporal drift of the interferometer’s optical path difference (OPD, Δ*L*). This was done using the Astro A313 interrogator while applying a DC voltage sweep from −5 V to +5 V. The voltage was held at each step for 140 s with an increment of 0.5 V, all under normal ambient (climatic) conditions.

Optical propagation losses were derived from the contrast of the interference pattern and the power of the maximum resonance peak in the reflected spectrum. These measurements employed a tunable laser source Keysight 81606A (USA), an optical circulator, and the integrated photodetector within the source, operating across the 1500–1600 nm band.

### 2.4. E-Sensor Calibration Technique

**Figure 2:**
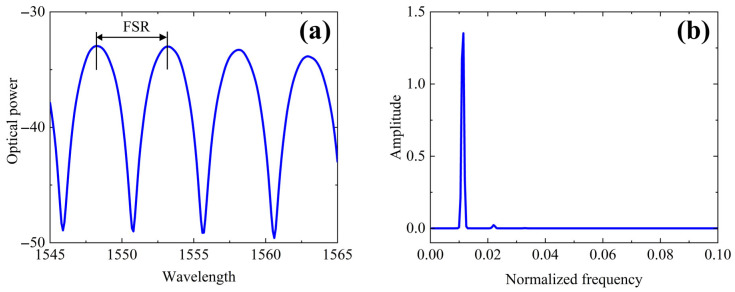
IOES typical data: reflection spectrum (**a**) and Fourier transform signal (**b**).

Typical interferograms (reflected spectra) and their corresponding Fourier transforms are shown in Figure 2. In such systems, the OPD is the target measurand. Several effective algorithms exist for demodulating the OPD (which corresponds to the frequency of the interference signal). These algorithms can be categorized into two types (referred to as TYPE 1 and TYPE 2), distinguished by their use of the interference signal’s phase information *φ* [31,32].

**Figure 3:**
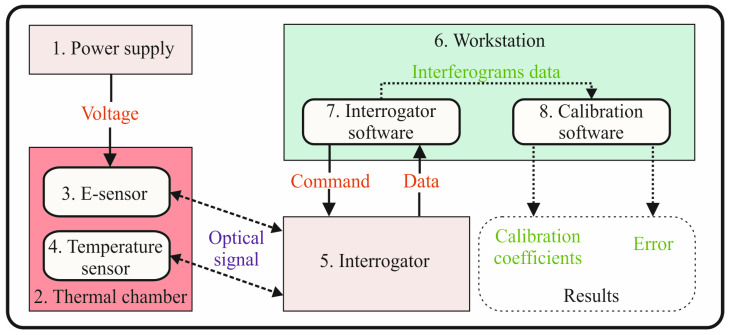
E-sensor calibration scheme: (1) power supply Gwinstek GPP-743234; (2) thermal chamber Mini. Incubator DSI-100D; (3) IOES sensor; (4) Fiber Bragg grating temperature sensor; (5) interrogator Astro A313; (6) laptop workstation; (7) interrogator software Astrosoft v2.13.1; (8) calibration software GS v1.4.

In the TYPE 1 demodulation algorithm, the phase *φ* is either unknown or deliberately ignored. Algorithms in this category are essentially equivalent to locating the interference peak position in the discrete Fourier transform spectrum. In contrast, TYPE 2 algorithms explicitly utilize the known or estimated phase *φ*. This approach provides a significant improvement in resolution by one to two orders of magnitude compared to TYPE 1 methods [32]. In this work, we employed a TYPE 2 algorithm. The experimental setup used to determine the IOES calibration function is shown in Figure 3.

The optical pathlength (cavity length) is estimated using the full (unwrapped) phase of the signal. The frequency estimate, obtained via Fourier analysis, serves to resolve the inherent ambiguity in the phase estimate. Subsequently, the phase estimate refines the measurement, achieving accuracy approaching the Cramer–Rao bound. As a result of this combined approach, the algorithm’s relative error does not exceed 0.1%. The corresponding potential absolute error is less than 0.05 nm.

The goal of calibration is to find a function *V*(*φ*) that maps the measured phase to an applied voltage with minimal deviation from the true voltage value [33]. This is achieved by determining the calibration coefficients for *V*(*φ*) through the minimization of the sum of squared residuals $\sum_{i=1}^{J}{\left(V_{b}\left(\varphi_{i}\right)-V_{i}\right)}^{2}:$

(9) $$V_{b}(\varphi )\;=a_{1}\varphi^{n-1}+a_{2}\varphi^{n-2}+\;\ldots \;+a_{n}.$$

The following parameters were used to assess the measurement error: maximum absolute error—∆*_max_*, mean absolute error—∆, maximum deviation—*γ_max_*, mean deviation—*γ*, standard deviation—*σ*.

(10) $$\Delta_{max}=max\left|V_{b}\left(\varphi_{i}\right)-V_{i}\right|,\;\forall i=1\ldots j,$$

(11) $$\underline{∆}=\sum_{i=1}^{J}\frac{{\left(V_{b}\left(\varphi_{i}\right)-V_{i}\right)}^{2}}{j}\;,$$

(12) $$\gamma_{max}=\frac{\Delta_{max}}{\max\left(V\right)-\min\left(V\right)}\cdot 100\%,$$

(13) $$\underline{\gamma }=\frac{\underline{∆}}{max(V)-min(V)}\cdot 100\%,$$

(14) $$\sigma =\sqrt{\frac{1}{J}\sum_{i=1}^{J}{\left(\Delta_{i}-\underline{∆}\right)}^{2}},$$

## 3. Results

### 3.1. E-Sensor Parameters

**Figure 4:**
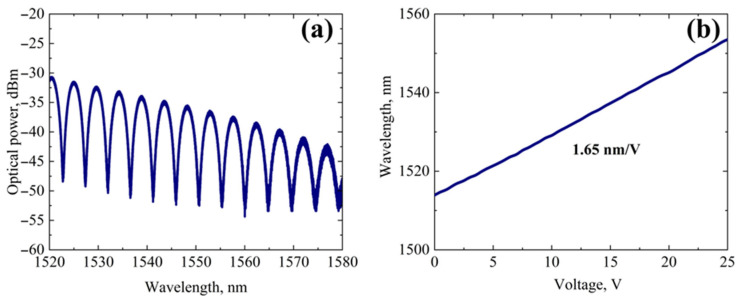
E-sensor parameters: (**a**) reflectance spectrum and (**b**) electro-optical sensitivity.

The reflection spectrum and the electro-optic sensitivity of the IOES across the 1500–1600 nm wavelength range are presented in Figure 4. The sensor’s parameters are determined by both the device geometry and the properties of the reduced LN substrate.

The measured electro-optic sensitivity of the sensor is approximately 1.65 nm/V. This value indicates that the interference spectrum shifts by 1.65 nm per 1 V change in the applied voltage. A higher electro-optic sensitivity allows for the detection of lower voltage levels for a fixed spectral resolution of the measurement system, thereby defining the sensor’s detection threshold and its suitability for weak electric field sensing. The LN reduction process involves a partial change in the valence state of Nb atoms (Nb^5+^ → Nb^4+^) [34]. Since the electro-optic properties of LN depend on the valence state of Nb, this reduction leads to a decrease in the maximum electro-optic coefficient *r*_33_ by approximately 13.6% compared to pristine LN [35]. For the IOES, this represents a deliberate compromise between achieving long-term stability (via reduction) and retaining sufficient electro-optic performance.

In the current implementation, the total optical insertion loss of the IOES is approximately 25 dB. This loss arises from the combined contributions of fiber-to-waveguide coupling loss, propagation loss in the APE waveguides, and loss at the dielectric mirror. While this level of loss necessitates the use of a sufficiently powerful optical source to maintain an acceptable signal-to-noise ratio, it does not constitute a fundamental limitation. Future work will focus on optimizing the APE waveguide parameters in reduced LN to achieve total losses below 10 dB.

The measured FSR is about 5 nm at 1550 nm, which is dictated by the physical path length difference between the interferometer arms. This spectral periodicity enables continuous tracking of the optical phase shift.

### 3.2. E-Sensor Instability

**Figure 5:**
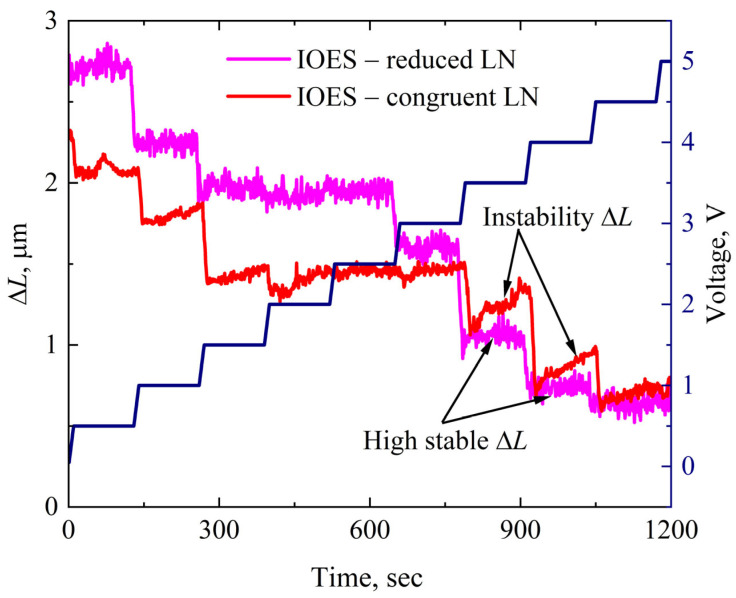
IOES stability based on reduced and congruent LN under applied voltage.

The inherent instability of conventional LN-based IOES originates from electrical inhomogeneities that form on the crystal surface due to its pyroelectric properties [28]. This instability is clearly observed in an IOES fabricated on congruent (non-reduced) LN when a constant voltage is applied (Figure 5). It manifests as a continuous drift in the optical path difference Δ*L* under fixed bias conditions. This drift is caused by the slow relaxation of surface charge inhomogeneities via various charge transport mechanisms, such as ion conduction and charge trapping at defect centers. These intrinsic fields superimpose on the externally applied electric field, thereby modulating the effective refractive index of the waveguide. Consequently, this phenomenon introduces systematic errors into the sensor’s calibration characteristics and degrades long-term measurement accuracy.

In stark contrast, the IOES based on thermally reduced LN demonstrates significantly enhanced stability. This improvement is achieved through the high-temperature reduction process, which fundamentally alters the crystal’s charge state. During reduction, the partial evaporation of molecular oxygen and lithium oxide breaks covalent bonds and induces a partial change in the valence state of niobium ions (Nb^5+^ → Nb^4+^) The resulting generation of free charge carriers (in the form of polarons) increases the electrical conductivity of the material by several orders of magnitude. This heightened conductivity effectively screens internal pyroelectric fields and provides superior stability against variations in external conditions, such as electric field and temperature.

Therefore, the use of thermally reduced LN enables the development of highly stable IOES with predictable and reproducible performance, making them suitable for reliable long-term operation in real-world applications.

### 3.3. E-Sensor Calibration

**Figure 6:**
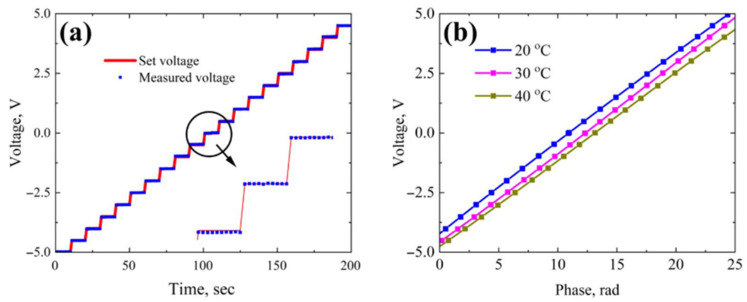
Calibration results: measured voltage at 20 °C (**a**) and phase dependence of voltage at three temperatures (**b**).

The IOES was calibrated by applying DC voltages ranging from −5 to +5 V in 0.5 V increments at a stabilized temperature of 20 °C under normal ambient conditions (Figure 6a). The optical phase was recorded at least ten times for each voltage step. The experimental data were fitted with polynomial functions, with coefficients ai determined according to Equation (9) for polynomial degrees of 1 (linear) and 3 (cubic). While the phase-to-voltage dependence *V*(*φ*) is well described by a linear function (Figure 6b), using a third-degree polynomial improves the measurement accuracy by approximately a factor of two for the reduced-LN-based IOES (see Table 1).

Table 1 summarizes the calculated measurement errors based on Equations (10)–(14). The maximum relative error *γ_max_* for the IOES based on reduced LN was 0.47%. According to the international standards IEC 61869-11 [36] and ISO/IEC Guide 99:2007 [37] (VIM), this corresponds to an accuracy class of 0.5, which represents a high precision level suitable for both laboratory and industrial applications. In contrast, the same analysis for an IOES fabricated on standard congruent LN yielded a significantly larger error of 2.6% (see Table 1) approximately five times higher than that of the reduced-LN device. This substantial discrepancy is attributed primarily to the pyroelectric effect in congruent LN. The increased surface conductivity achieved through thermal reduction enhances optical stability and effectively reduces the fundamental sensor error.

To the best of our knowledge, the typical relative error reported for electric field sensors based on the Pockels effect ranges from 3% to 10% [19,38,39]. The IOES presented here demonstrates superior accuracy compared to these existing devices. Furthermore, we anticipate that the baseline relative error can be further reduced through optimization of the optical design and implementation of active thermal compensation.

Acknowledging the influence of temperature on sensor performance, we conducted additional calibration measurements at 30 °C and 40 °C. The resulting calibration curves are plotted together with the 20 °C data in Figure 6b. These curves clearly illustrate a significant temperature dependence, confirming that thermal compensation will be essential for operation in real-world environments. The results also reveal a nonlinear shift in calibration with temperature.

In summary, an IOES based on thermally reduced LN was successfully fabricated, characterized, and calibrated. The results confirm its high potential for practical deployment and indicate clear pathways for further improvement in accuracy and environmental stability.

## 4. Conclusions

This work demonstrates the successful development and characterization of a highly stable IOES based on thermally reduced LN. The sensor, implemented in a Michelson interferometer configuration, exhibits an electrooptic sensitivity of 1.65 nm/V, an optical insertion loss of 25 dB, and FSR of 5 nm at 1550 nm.

The key advantage of this approach is the drastic suppression of operational instability under applied bias, achieved by mitigating the intrinsic pyroelectric effect of LN through thermal reduction. Calibration of the IOES yielded a maximum relative error of 0.47%, which corresponds to a high precision accuracy class of 0.5 according to the international standard IEC 6186911, making it suitable for demanding industrial and laboratory applications.

These results establish thermally reduced LN as a highly promising material platform for enhancing the long-term stability and reliability of a broad range of electrooptic devices. Looking forward, we aim to further improve the sensor’s performance by targeting a relative error of 0.1–0.3%. The planned optimization includes reducing the total optical insertion loss to below 10 dB, implementing and testing an active thermal compensation scheme, developing a dedicated demodulation algorithm for thermal stabilization, AC dynamic performance characterization, and conducting comprehensive long-term stability tests.

## Figures and Tables

**Table 1:** IOES measurement errors.

**Reduced LN**					
**Function**	**Error, V**		**Error, %**		***σ*, V**
	**∆*_max_***	**Δ**	***γ_max_***	** *γ* **	
Linear	0.11	0.04	1.05	0.36	0.02
3rd rank polynomial	0.05	0.02	0.47	0.17	0.01
**Congruent LN**					
Linear	0.28	0.16	2.81	1.65	0.07
3rd rank polynomial	0.26	0.16	2.60	1.65	0.06

## Data Availability

The data that support the findings of this study are available from the corresponding author upon reasonable request.
